# Perspectives and Attitudes towards the Functional and Safety Aspects of Seaweeds for Edible Applications in India

**DOI:** 10.3390/foods10123026

**Published:** 2021-12-06

**Authors:** Tejal K. Gajaria, Vaibhav A. Mantri

**Affiliations:** 1 Division of Applied Phycology and Biotechnology, CSIR—Central Salt & Marine Chemicals Research Institute, Gijubhai Badheka Marg, Bhavnagar 364002, India; tejalgajaria@gmail.com; 2 Academy of Scientific and Innovative Research (AcSIR), Ghaziabad 201002, India

**Keywords:** antioxidants, bioactive, consumer attitude, functional foods, gastronomy, macroalgae

## Abstract

Seaweeds are inevitable resources of nutrition bearing favorable rheological characteristics, which has resulted in their inclusion in a variety of daily consumer products. India, with its vast coastline and over 1000 species of seaweeds, presents tremendous potential to bring this resource into nutraceuticals and the food sector. The present survey was designed for the Indian population, which was further classified according to diet preferences, age groups, gender and various occupations. Their perceptions regarding nutritional aspects, sensory views, safety hazards and resource reliability were recorded. Among all groups studied, gender represented significant differences upon the various safety opinions recorded (*p* < 0.001) compared to the occupations, age groups and diet preferences studied. In addition, the dataset revealed the pro-phycological behavior of consumers subjected to vital concerns about bioresource reliability and pre-processing to avoid health hazards related to wild harvest or on-shore cultivated samples. In addition, consumer responses also revealed potential inhibitory factors in edible applications such as taste and smell. This study suggests that collaborative efforts among media, culinary experts and phycologists could play a pivotal role in promoting seaweeds in the rapidly expanding food sector industry of India.

## 1. Introduction

Seaweeds are marine, renewable resources of the world’s oceans, immensely contributing towards ecological services, the economy and society, yet they are under-utilized. They are ubiquitous in occurrence and distributed throughout the world, from the Arctic as well as Antarctic to tropical equatorial seas. They have been harvested by coastal populations for centuries for domestic purposes mainly as food, feed and agriculture [1]. Pre-historic evidence suggested the use of seaweed as a human food in Japan [2] and Korea [3]. The use of seaweed outside Asian countries has been less prevalent; however, the Marquesas Archipelago (French Polynesia), Norway, Ireland and Scotland present historical records [4]. The globalization of markets coupled with innovations in food technologies has changed food patterns drastically in several parts of the world. Studies have confirmed bountiful and rich sources of trace elements, minerals, polyunsaturated fatty acids, vitamins, iodine, carbohydrates and proteins from them. Further, the realization of benefits from consuming seaweeds due to the presence of functional ingredients and medicinal compounds introduced them in the form of a macrobiotic diet to the Western world [5]. The consumption of seaweed in the form of sushi as well as an ingredient in snack foods has experienced significant growth in recent times in several countries, including India, Norway and Australia [6,7,8]. The use of seaweed and seaweed-derived products is on the rise globally. Their vegetarian origin, sustainable production technologies and proven accrued health benefits make seaweeds a highly innovative enterprise and ideal candidate for the establishment of small businesses to cater to local needs.

The last report commissioned by the World Bank, titled ‘Seaweed Aquaculture for Food Security, Income Generation and Environmental Health in Tropical Developing Countries’, advocated the urgent need to utilize seaweed biomass for human food and animal feed. The human population is estimated to reach 10.9 billion globally by 2100 [9]. It has been estimated that burgeoning global population growth necessitates the production of 50–70% additional food by 2050. Nevertheless, globally 32.39 million tons fresh weight of seaweeds are being produced, with an associated economic value of USD 13.3 billion, which is expected to reach 22.13 billion by 2024 [10]. The estimations reported the availability of approximately 48 million km^2^ suitable seaweed farming areas spanning over 132 countries, of which only 0.001% is being used by about 37–44 nations [11]. This has reinforced the fact that the estimated production of 500 million tons dry weight can be achieved by 2050 [9]. Thus, seaweed farming and developing seaweed-based edible products are the two critical components in achieving both blue economic development as well as sustainable developmental goals at the regional level. 

Along India’s long coastline of about 7500 km, the natural seaweed collection has been the source of livelihood for coastal fisherwomen for several decades, especially in Gujarat and Tamil Nadu. Seaweed-based processing industries utilize this feedstock for the production of hydrocolloids, mainly agar, alginate and to a lesser extent carrageenan [12,13,14]. The use of seaweed for food has been considered as an area of research by CSIR—Central Salt and Marine Chemicals Research Institute, Bhavnagar. Conscious efforts have been made to enumerate protein, amino acids and peptides from seaweeds [15]; amino acids, minerals, proteins, pigments and lipids [16,17]; and iodine [18], besides developing a cultivation protocol for several species [19,20]. Recently, our group has developed an integrated method of extraction of crude proteins with the recovery of mineral-rich sap, lipids, ulvan and cellulose from the fresh biomass of *Ulva lactuca* [21]. Nevertheless, these studies have established the proof of concept that seaweed-based products have an advantage to be deployed and marketed in India as a commodity product, where a staggering 31% of the population (over 350 million people) are pure vegetarians. The food industry in India is worth around USD 155 billion, which is expected to reach about USD 344 billion by the year 2025, with an annual increase of about 4.1% [22].

To the best of our knowledge, nothing is known about the opinion of the general public regarding the utility of seaweed as a food in India. It may be noted that the un-familiarity of such new products in the edible food sector to the consumer might be intimidating, and the acceptance or non-acceptance of such a product might be crucial for the success of the industry. Empirical data in the form of academic literature for seaweed consumption in Western societies are seldom available. Prager (2017) has reported the acceptance of seaweed as a viable source of a complete protein based on consumers’ perceptions based on the literature. It may also be noted that few studies have elicited the attitudes of consumers towards including seaweed in a Nordic diet based on sensory analysis [23] and profiled consumers who are likely to eat seaweed products in Australia [24]. The Government of India under its fisheries revitalization plan considers seaweed as a strategic commodity other than shrimp and tuna. The policy intervention and generous funding support under Pradhan Mantri Matsya Sampada Yojana (PMMSY), Blue Economy (BE), and Fisheries and Aquaculture Infrastructure Development Funds (FIDF) has gained ground for establishing seaweed-based industries in the country with special emphasis on the niche sector of edible seaweeds. In addition, with favorable policy systems in the food processing industry sector, which is growing with an average annual growth rate of 8.41%, the Indian food market size is expected to reach USD 544 B by 2020–21 [22]. It would therefore be worth recording the level of awareness, adaptation interests and knowledge base of a targeted audience in India pertaining to seaweeds as an edible source. These details are expected to provide fundamental grounds to obtain consumer perceptions towards novel food resources, which are essential to build strategic planning for branding and promotions.

## 2. Materials and Methods

### 2.1. Survey

The survey was developed to determine the awareness and adaptation interests of a targeted audience in view of collecting a preliminary profile of participants. The survey was divided into five sections: Section 1 Introduction regarding the study and potential role of participants. Section 2 Personal details such as age group, gender, occupation/present activity and diet preferences. The Section 3 contained 11 questions regarding their fundamental understanding associated with seaweeds and whether they agree to the link between seaweeds and health. Section 4 comprised 10 questions to ascertain the safety concerns regarding seaweed sources, and Section 5 contained 15 statements with graded choices starting from strongly agreeing to strongly disagreeing pertaining to the environmental consciousness of the participants (Supplementary Table S1). The form was materialized in the form of Google Forms and circulated among the public via a generated link, authentication of participants was confirmed by obtaining valid email ID to avoid any spam autofilling and respective responses of participants were sent back to their respective email ID provided at the beginning of survey for record. In addition, the link was forwarded to various academic departments to ensure maximum participation. The survey was carried out up to 30 days from the first distribution of forms followed by deactivation of the link before starting the processing of data. The data were saved, managed and processed using Microsoft Excel version 2016. The population was divided per diet preference, i.e., strictly vegetarian (SV; people that utilize only vegetarian sources for food and nutrition), strictly non-vegetarian (SN; people that utilize only meat products as sources of food and nutrition), partly non-vegetarian (PN; people that utilize both vegetarian as well as non-vegetarian sources for food and nutrition) and eggetarian (EG; people that utilize eggs together with vegetables but do not eat any meat).

### 2.2. Statistical Analysis

The survey was conducted starting from 10 June 2020 and closing on 25 June 2020. The Google Forms link was distributed randomly via email and various social media platforms. A total of 310 responses were obtained during the 15-day period. The database was maintained as well as statistically analyzed in Microsoft Excel, 2016 and distributed per age group, occupation, gender and diet preference. Significant differences of means among groups were determined using ANOVA in addition to Student’s t-test for comparison between two samples. The survey data were analyzed by assigning all positive responses a numerical value of ‘2′ and negative responses a value of ‘1′, so that the obtained mean (1 < µ < 2) could be assigned either towards positive or negative following Simha et al., (2018) [25]. To estimate environmental awareness and attitudes regarding the environmental conservatory responsibility of participants, individual responses for New Environmental Paradigm (NEP) statements comprising 15 questions were provided at the end of the seaweed section. The responses were obtained using a scale of 1 to 5, with 1 indicating strongly agree and 5 strongly disagree (Supplementary Table S1). The average NEP rating (1 < µNEP < 5) of the dataset was calculated for all questions at individual levels. An average NEP rating of 3 was considered the point between pro-ecological and anthropocentric environmental views.

## 3. Results

### 3.1. Consumer Attitudes to Seaweed Safety and Utilization 

Individual consumer attitude scores varied considerably among groups. The overall attitude score of the dataset was found to be 1.20 ± 0.16, which is slightly negative. The scores (Table 1) suggest that the consumers were either devoid of information regarding safety concerns and aspects of seaweeds or required further analysis to pull their opinion. Segregated attitude scores of the consumers based on different categories of the demographic variables suggested that: (a) there was no exact correlation between age categories and attitude as the acceptance of seaweeds in food seemed dependent on the basis of their pre-treatment; (b) female respondents were slightly more negative than their male counterparts (1.20 vs. 1.24; *p* < 0.0004); and (c) the overall score of dietary preferences did not show any significant difference of opinions.

When different weights were assigned to the four aspects assumed to determine consumer attitude, the overall attitude score was changed (Table 2). When more importance was assigned to the safety aspects and pre-treatment prior to seaweed consumption compared to those of bioactive potential and passive immunity development, the overall score decreased from 0.53 ± 0.18 to 0.32 ± 0.13, with *p* value < 0.0001. This explains that the population score is more inclined towards learning more about the competitive microbial pathogens and heavy metal contaminations the seaweeds may have compared to the pre-treatment and bioactive potentials of seaweeds. 

### 3.2. Attitude Towards Safety and Utilization: Environmental Outlook

The New Ecological Paradigm (NEP) scale, which is sometimes referred to as the revised NEP, is a survey-based metric devised by the US environmental sociologist Riley Dunlap and colleagues. It is designed to measure the environmental concern of groups of people using a survey instrument constructed of fifteen statements. Respondents are asked to indicate the strength of their agreement or disagreement with each statement. Responses to these fifteen statements are then used to construct various statistical measures of environmental concern. The NEP scale is considered a measure of environmental world view or paradigm (framework of thought). The NEP rating in the present study ranged from 1 for strongly agree to 5 for strongly disagree. The mean NEP rating of the dataset was 2.38, indicating that the respondents were environmentally conscious and in favor of ecological conservation (Table 3). In particular, the majority endorsed the possibility of an eco-crisis, as well as anti-anthropocentric views, but mixed responses were obtained in the facet of the balance of nature and for the view of the capacity of Earth to cope with drastic environmental changes. 

### 3.3. Safety Perspectives and Seaweed Utilization

**Figure 1:**
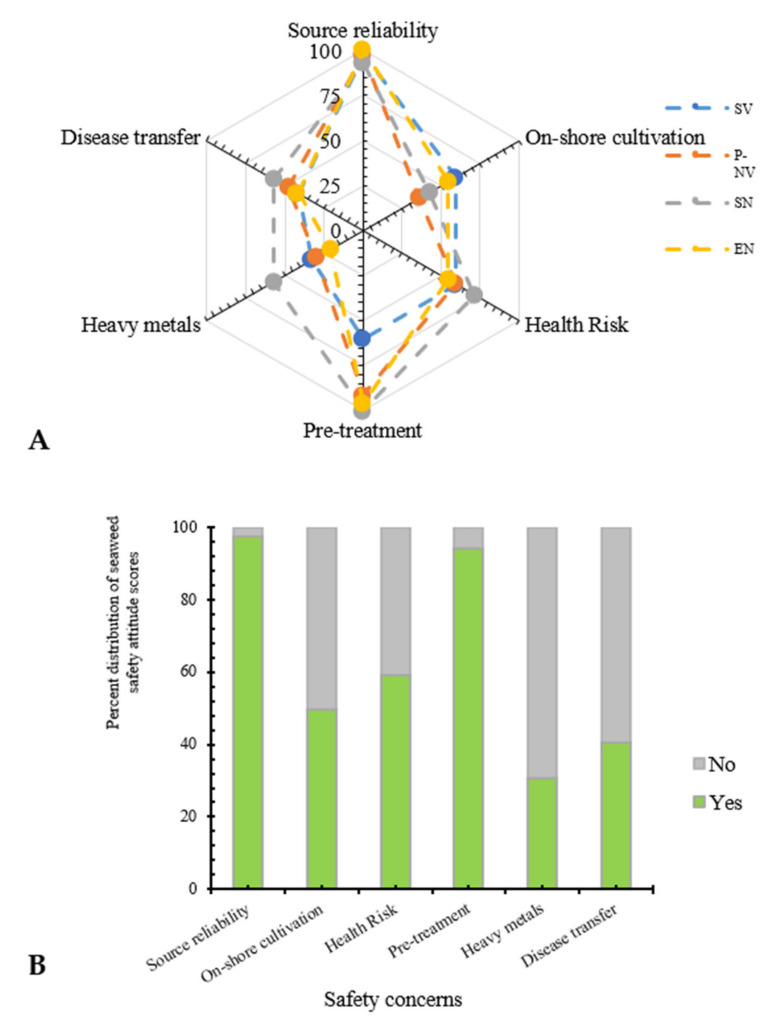
(**A**) Influence of diet preference upon consumer attitude score towards safety aspects of seaweeds; (**B**) Overall impression of respondent distribution attitude score towards safety aspects of seaweeds.

In the present study, safety concerns related to the adaptation of seaweeds as a nutritional supplement were analyzed (Figure 1A,B). Irrespective of diet preference, the participant audience unanimously showed concerns regarding the origin/sources of seaweeds being utilized for edible applications (*p* < 0.05 based on gender responses; Table 1). Moreover, highly encouraging responses, i.e., 94.5%, were obtained towards the possible adaptability of pre-treated seaweeds prior to release in the market. In addition, 59.2% and 40.7% of responses believed that seaweeds might be associated with potential health risks as they grow in the open sea and might be a possible route to microbial infections or disease transfer, respectively. In addition, although the bioremediation potency of seaweeds has been well-documented worldwide, only 30.8% of participants believed their possible implications upon health risks, whereas 59.5% opted for further information regarding this subject prior to making their opinion, and this group was comprised of 42.9% strictly vegetarian (SV) participants. Interestingly, when these responses were further classified based on the various occupations studied, homemakers/housewives scored highest (100%), followed by the self-employed (71.4%) and higher education students, i.e., PhD/MS/MD (68.4%), for opting for further analysis pertaining to the possible heavy metal-associated health risk posed by seaweeds. This trend shows an attribute of the dependence of participants on published/available literature and hence the responsibility prior to establishing an understanding about the nature of seaweed. However, in continuation with the heavy metal- and microbial pathogen-associated health risks of seaweeds, 62.6% of responders (with highest votes from SV: 71.5% followed by 66.6%, 57.7% and 50% for SN, PN and EV, respectively) believed off-shore cultivation could be a favorable resource for seaweeds intended for edible applications.

### 3.4. Understanding Various Groups

#### 3.4.1. Diet Preference 

**Figure 2:**
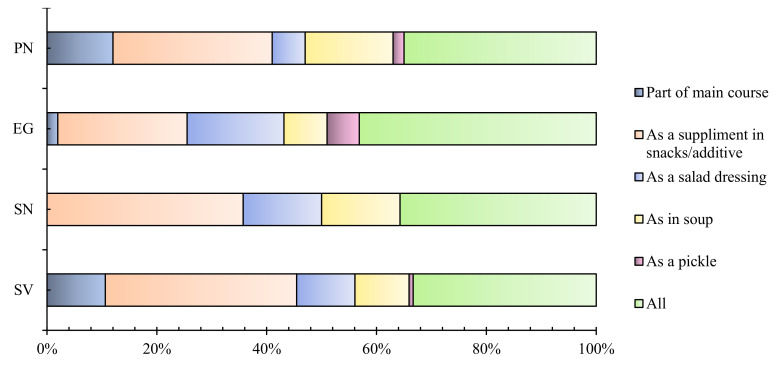
Consumer preferences over inclusion of seaweeds in their diet.

Furthermore, safety points classified according to diet preferences showed all groups agreed with the source reliability concerns. In addition, except the strictly vegetarian (SV) group, all groups variably agreed (59.3%, 35.9%, 42.9%, 54.7% for SV, PN, SN, EG, respectively) that the reason for resource reliability might be due to its co-culturing with other marine organisms regarding on-shore cultivation or wild harvesting. Figure 2 shows various preferences of consumers upon seaweeds in their respective diets. As the figure illustrates, all diet groups including the strictly vegetarian population prefer to have seaweeds as a supplement or additive in snacks (34.8%) compared to other options such as in salad dressings (10.6%), soup (9.8%), pickles (0.75%) or main courses (10.6%). In addition, alternatives, i.e., as a pickle or in main courses, were one of the least selected options, which might be due to the unawareness or neophobia of seaweeds and related products, specifically regarding vegetarian populations who perceive that most seaweed-based main course recipes include either fish or meat products. However, a significant population from the SV group (33.3%) chose multiple options for seaweed inclusion in their diet compared to EV (43.1%), SN (35.7%) and PN (35%).

#### 3.4.2. Age Distribution 

**Figure 3:**
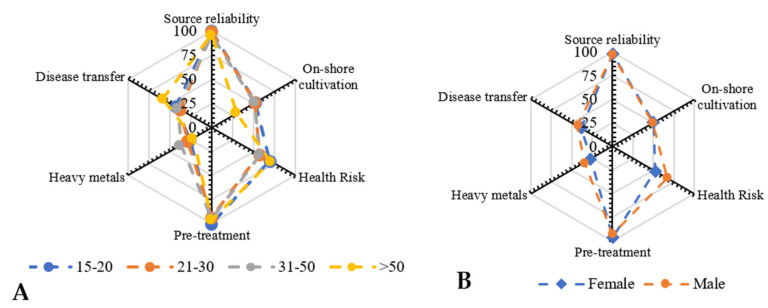
(**A**) Consumer safety concerns based on age group and (**B**) gender.

The distribution of responses based on gender, age group and occupation further refined the understanding among each group. In the present study, we have included young students (15–20 Y) to retired people (>50 Y) to gain insights regarding the impact of the experience of aged people versus young minds pursuing either academic jobs or higher education at various levels (Figure 3A). The results show strong agreement among the various age groups on their opinion regarding seaweed source reliability and pre-treatment prior to consumption, as in the case of diet preference. However, the majority of experienced (>50 Y) people believed seaweeds might be associated with heavy metal contamination due to their absorption efficiency, whereas other groups, i.e., 15–20 Y, 21–30 Y and 31–50 Y, believed that the association of seaweeds with other marine life forms may limit their choice to prefer them as a food source (*p* = 0.99; F_crit_ = 3.09).

#### 3.4.3. Gender-Based Perspectives 

On the other hand, there was no statistically significant gender bias upon seaweed safety concerns (*p* = 0.82; F_crit_ = 4.96). In addition, the responses were unanimous in the case of seaweed source reliability, biomass pre-treatment and their on-shore cultivation, whereas the probability of disease transfer and heavy metal absorption showed a marginal difference with the highest difference on the health-associated risks (Figure 3B).

#### 3.4.4. Occupational Differences

**Figure 4:**
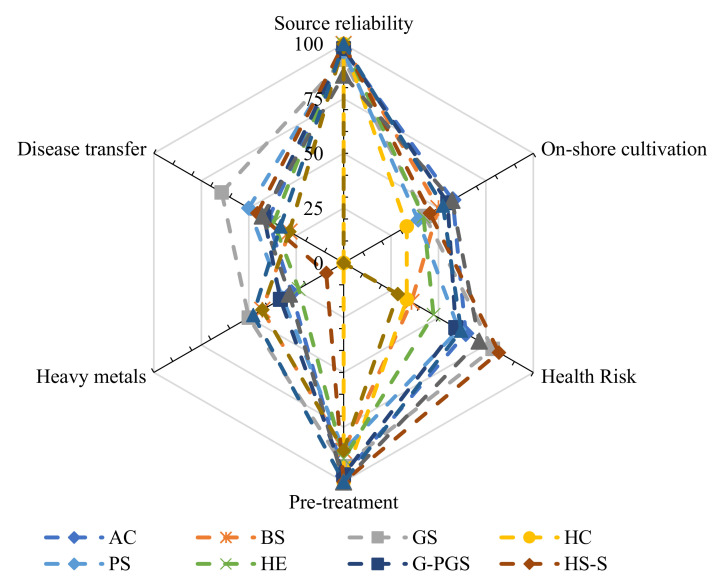
Safety response distribution of participants based on their respective occupations.

Out of the 12 different groups studied, source reliability and the pre-treatment of biomass remained one unanimously agreeable point, but they differed largely on the health-associated risks and the possibility of disease transfer (*p* = 0.95; F_crit_ = 2.00) (Figure 4). In addition, graduate students showed maximum concerns regarding all the safety aspects studied compared to home care personnel and higher secondary students presenting their enthusiasm to adapt this novel food source.

## 4. Discussion

Although there are several edible seaweeds reported from the Indian shore that are suitable for the preparation of popular products, namely salad, jam, jellies, curry and porridges, they are not yet incorporated in restaurant meals or local cuisines of the general populace [26]. The flavors of popular seaweed dishes from Japan or other Southeast Asian nations are too delicate for Indian people and thus new seaweed recipes were developed that can suit the Indian taste and palate. The sensory evaluation of fish cutlets containing *Eucheuma* powder by panelists revealed that the incorporation of up to a 10% level of seaweed gave the product an acceptable quality [27]. The current push from the Ministry of Fisheries as well as the Animal Husbandry, Dairy industries together with various food companies is towards integrating seaweeds in local food courses, which necessitated the documentation of Indian consumer perceptions towards seaweed as a food product. The understanding obtained would allow emerging start-ups to gain valuable insights into the preferences and gap areas of prospective consumers to design and develop effective marketing strategies.

Overseas brands entering the edible food sector have propelled gourmet snack food culture in India. This scenario has resulted in consumers being willing to experiment with new products that are high in health benefits and taste. It may be noted that, with the trend of growing health awareness among consumers, the entry of seaweeds into the edible market is inevitable. The key finding of this study suggests that participants from all clusters, i.e., age group, gender, diet preferences and occupations, unanimously showed great concerns about the origin and safety of seaweed biomass pertaining to their habitats and bioaccumulation potentials regardless of their nutritional output. These observations are in accordance with recent studies conducted in Italy and Australia, wherein consumers already had previous exposure to seaweeds, i.e., 57% and 74%, respectively [24,28]. However, in the present study, despite 68.2% of participants being aware of seaweeds, only 57.9% possessed previous knowledge about their nutritional benefits. In addition, 71.5% of the population showed interest in or willingness to know more about these nutritionally rich seaweeds, and the enthusiasm for perceiving additional knowledge about their nutritional potential was reported to be equally spread between males (80.1%) and females (78.8%), which is in contrast to the study reported by Birch et al., (2018), wherein females (42%) reported a higher level of consumption of seaweeds compared to males (32%). These findings are in accordance with Al-Thawadi (2018) and Bührlen et al., (2005) regarding the higher acceptance of edible seaweeds by the public, which might be due to their proven nutritional benefits as well as their emerging role in the area of complementary and alternative medicines [29,30,31]. Furthermore, the major form of seaweed utilization was found in the form of snack items followed by soups, similar to the study of Birch et al., (2018). In addition, a study based on motivational differences in food orientation conducted by de Boer et al., (2013) in the Netherlands showed that consumers with a higher level of education, urban background, with highly involved or innovative minds and taste-oriented respondents appreciated seaweeds as an alternative source of proteins compared to other meat alternatives, which is partly similar to the results of the present study [32].

A strong positive response was obtained when alternatives such as off-shore cultivated and pre-processed biomass were suggested as 90.9% of participants agreed when off-shore cultivation and (94.7%) the pre-processing of biomass were presented as an alternative to wild harvested biomass. These factors suggest strong concerns and beliefs among participants regarding the safety and processing of biomass to avoid any health-related risks that are identical to ones reported in the Italian as well as Australian study [24,28], wherein health and nutritional benefits were the most important driving factors towards voting for seaweeds as essential foods. Such concerns should be advocated by proper marketing campaigns with sufficient labeling regarding the nutritional benefits as well as safety aspects of products such as microbial load, heavy metal contents, etc. However, potential agreement was observed when alternatives were presented to consumers against possible resistances that indicate Indian consumers perceive seaweed consumption to be more associated with its health benefits where sensory properties and safety precautions might be duly addressed. Similar conclusions were obtained from the Italian and Australian studies that more information about seaweed characteristics such as taste and nutritional benefits might be the potential drivers for the sustainable development of this market in the future [24,28]. In addition, as per [33], public intervention could be a very effective way of handling neophobia and misunderstanding regarding the risk factors associated with seaweed consumption. Timely campaigns and workshops could play great role in familiarizing such resources to neophobic or sensitive populations. Unlike Birch et al., (2018), we did not find the enthusiasm for edible seaweeds limited to a particular age group or gender, wherein the larger population was among the age of 21–30 (*n* = 165) followed by 31–50 (*n* = 94) and >50 (*n* = 27). However, in agreement with Palmieri and Forleo (2020), institutional intervention is necessary to bring about the essential facts regarding long-term effects of seaweeds on health and well-being for humans in the public domain, which would strengthen the possibility of seaweeds as a part of routine diet and would also encourage public involvement to overcome any fears regarding acceptance or risk factors associated with seaweed consumption. The upcoming key limiting factor might be the affordability and sustainability of a seaweed-based product in the market. Cost-effectiveness largely depends on the extent of pre-processing required to formulate the end product, which demands a user-experience-based design that not only represents the quality of the product but should be able to convince consumers to buy it. Such targets require a thorough market analysis in addition to studying the purchasing power of the community, which can help to serve a diverse choice of products with a wide range of functionalities.

In the present study, 45.3% (*n* = 140) of participants followed a strictly vegetarian diet and they largely believed in edible applications of seaweeds as a supplement to snack items. It is also evident from the data that among all vegetarian consumers, 44.2% would have issues with either the smell, taste, source reliability or a combination of any of these factors as a barrier to directly including seaweeds as a main course meal. We suggest, in accordance with the previous study by [34], that product formulation should be aligned with the sensory perceptions of consumers in addition to advocating their nutritional benefits. Furthermore, developments into creative gastronomy involving chefs and phycologists are instrumental for break-through developments in the area of “phycogastronomy” [35]. This study unequivocally brought the facts that Indian consumers reported unique habits and thus their preference towards incorporating seaweed-based products in their diet. Retailers and manufacturers should incorporate this marine renewable source by mainly targeting the vegetarian population. This can be performed without compromising on taste considering the younger generation, while health benefits for older people should be emphasized to ensure success. Further, this study is helpful for creating a framework of a broad strategic business structure and boosting preparedness.

## 5. Implications 

The present study is a one-of-a-kind attempt regarding the awareness, perspectives and personalized views upon the inclusion of seaweeds in the Indian cuisine, which revealed enthusiasm as well as the possible roadblocks for wider consumer acceptance. To improve upon the acceptance of seaweeds, effective communications and diverse marketing strategies are instrumental for targeting audiences. As evident from previous studies that consumers perceive food quality and food safety as interlinked concepts, developing products based on various nutritive requirements, preferred type of usage and environmental labeling would be convenient to draw attention from educated communities [36,37]. In addition, for targeting the vegetarian population, the major concerns remaining are taste, smell and textural properties and hence require a thorough analysis upon effective bioprocessing and product formulation strategies for wider acceptance among audiences. 

## 6. Limitations and Future Perspectives

This study is confined to 309 participants from the Indian population, but with India being culturally diverse and representing 28 states and 8 union territories, future research involving a larger sample size with maximum inclusion of culinary diversities would further expand and strengthen the understanding. The sensory properties of seaweeds such as smell and their raw taste are one of the long-standing barriers towards the acceptance of seaweeds in the Indian population, hence focusing on nutritional properties alone will probably not be enough to gain public acceptance. In addition, India has been steering towards seaweed cultivation of very limited species, with the majority targeted for hydrocolloid production for commercial purposes. The cultivation of seaweeds for edible applications is at a very nascent stage in India; the key factors might be (1) poor marketing strategies and (2) geo-climatic variability mostly due to the structural diversity in coastal geography and climatic conditions. These might be the rate limiting factors for the promotion and sustainability of this sector in the area. Hence, strategic planning involving governmental bodies and stakeholders remains pioneering in the growth of the seaweed sector in India. Affordability is another challenging factor for introducing edible seaweeds in India, wherein cost-effectiveness amidst cultivation, processing and packaging is a huge input largely causing higher product costs.

The objective of this study was to explore consumer awareness, attitude and adaptive perceptions regarding edible seaweed in India. This understanding would help in profiling consumer requirements and their fundamental issues, which would allow us to group them into clusters in order to devise potential marketing strategies. In addition, positive consumer attitude scores create a strong possibility for developing a market niche for edible seaweeds in Indian markets. Our findings show positive willingness regarding seaweed utilization not only as a food but also by the view of a nutritionally rich alternative with long South Asian culinary history. The Indian population is known for relatively faster adaptation rates in terms of experiencing a wide variety of cuisines including Western diets rich in cheese, Chinese, Italian, Thai, Mexican, etc. As India is comprised of >40% vegetarian population, the major safety and sensory concerns noted were the origin of biomass and the umami taste; however, the receptive responses towards biomass production using off-shore cultivation suggest a multi-directional growth opportunity in this sector, which also might serve towards the climate change mitigation goals of India. Collective information gathered in this article shows the tremendous growth potential of the seaweed industry in terms of cultivation, down-stream processing and product formulation, which require further refinement via major agricultural reforms to be sustained in this area.

## Figures and Tables

**Table 1:** The mean values of scores as represented for each group per safety question analyzed (* *p* < 0.05).

	Risk Factor		Pre- Treatment		Heavy Metal Absorption		Association with Pathogens		Inclusion into Food		Good for Passive Immunity		Overall	
Occupation	μ	*p*	μ	*p*	μ	*p*	μ	*p*	μ	*p*	μ	*p*	μ	*p*
Academic/educational duties, i.e., Teachers/Lab. Assistant/Assistant Prof./Associate Prof./Professor	1.28	0.40	1.34	0.22	0.63	0.40	0.71	0.23	1.60	0.44	1.53	0.95	1.18	0.91
Business	0.78		1.00		0.71		1.29		1.86		1.86		1.25	
Government service	1.64		1.50		1.14		0.93		1.36		1.43		1.33	
Homecare/homemaker	0.66		0.67		0.00		0.33		1.00		0.67		0.56	
Other	1.28		1.52		0.95		0.76		1.38		1.38		1.21	
Private sector service	1.21		1.36		0.86		0.96		1.54		1.50		1.24	
Pursuing higher studies, i.e., PhD/MS/MD	1.03		1.21		0.55		1.24		1.76		1.63		1.24	
Retired person	0.57		1.86		0.86		1.14		1.71		1.71		1.31	
Self-employed	1.43		1.14		0.57		0.71		1.71		1.43		1.17	
Undergraduate or postgraduate students	1.20		1.56		0.73		0.91		1.44		1.57		1.24	
Taking secondary or higher secondary education	1.64		1.45		0.45		1.09		1.64		1.73		1.33	
**Age group**														
15–20	1.39	0.42	1.39	0.15	0.61	0.42	0.96	0.48	1.22	0.09	1.35	0.62	1.15	0.72
21–30	1.20		1.32		0.73		0.88		1.64		1.55		1.22	
31–50	1.12		1.52		0.69		0.89		1.48		1.60		1.22	
50 and above	1.44		1.52		0.74		1.19		1.59		1.59		1.35	
**Gender**														
Female	1.23	0.0059 *	1.34	0.34	0.75	0.02 *	0.84	0.11	1.55	0.84	1.50	0.19	1.20	0.0004 *
Male	1.19		1.47		0.67		1.00		1.55		1.61		1.25	
**Diet preference**														
Eggetarian and Vegetarian	1.19	0.68	1.35	0.61	0.60	0.091	0.79	0.36	1.52	0.81	1.50	0.13	1.16	0.38
Partly non-vegetarian	1.25		1.33		0.83		0.84		1.56		1.51		1.22	
Strictly non-vegetarian	1.14		1.14		0.43		1.07		1.43		1.14		1.06	
Strictly vegetarian	1.19		1.50		0.70		1.01		1.57		1.64		1.27	

**Table 2:** The consumer attitude scores and modified attitude scores according to groups provided.

		n	Attitude Score	Modified Attitude Score
**Occupation**	Academic/educational duties, i.e., Teachers/Lab. Assistant/Assistant Prof./Associate Prof./Professor	73	1.18	0.30
	Business	14	1.25	0.35
	Government service	14	1.33	0.37
	Homecare/homemaker	3	0.56	0.13
	Other		1.21	0.33
	Private sector service	28	1.24	0.34
	Pursuing higher studies, i.e., PhD/MS/MD	38	1.24	0.34
	Retired person	7	1.31	0.36
	Self-employed	7	1.17	0.30
	Undergraduate or postgraduate students	93	1.24	0.33
	Taking secondary or higher secondary education	11	1.33	0.34
**Age group**	15–20	23	1.15	0.32
	21–30	165	1.22	0.32
	31–50	94	1.22	0.33
	50 and above	27	1.35	0.35
**Gender**	Female	157	1.20	0.30
	Male	152	1.25	0.35
**Diet preference**	Eggetarian and Vegetarian	52	1.16	0.30
	Partly non-vegetarian	103	1.22	0.31
	Strictly non-vegetarian	14	1.06	0.36
	Strictly vegetarian	140	1.27	0.34

**Table 3:** Environmental outlook of survey population.

NEP	Frequency					μ	σ	Facet
	SA	A	NA	DS	SD			
We are approaching the limit of the number of people the Earth can support.	124	62	70	28	22	2.22	1.27	Limits to growth
Humans have the right to modify the natural environment to suit their needs.	41	26	53	50	136	3.70	1.44	Anti-anthropocentrism
When humans interfere with nature it often produces disastrous consequences.	201	48	17	13	27	1.75	1.27	Balance of nature
Human ingenuity will ensure that we do not make the earth unlivable.	86	80	89	25	26	2.43	1.22	Balance of nature
Humans are severely abusing the environment.	191	61	22	9	23	1.73	1.19	Eco-crisis
The Earth has plenty of natural resources if we just learn how to develop them.	184	74	24	12	13	1.69	1.06	Limits to growth
Plants and animals have as much right as humans to exist.	236	33	17	8	13	1.47	1.01	Anti-anthropocentrism
The balance of nature is strong enough to cope with the impacts of modern industrial nations.	84	44	72	68	38	2.78	1.38	Balance of nature
Despite our special abilities, humans are still subject to the laws of nature.	189	59	37	15	7	1.67	1.02	Anti-exemptionalism
The so-called “ecological crisis” facing humankind has been greatly exaggerated.	62	68	81	38	57	2.87	1.38	Eco-crisis
The Earth is like a spaceship with very limited room and resources.	114	58	62	36	36	2.42	1.39	Limits to growth
Humans were meant to rule over the rest of nature.	30	22	40	45	169	3.98	1.36	Anti-anthropocentrism
The balance of nature is very delicate and easily upset.	79	68	75	55	29	2.63	1.30	Balance of nature
Humans will eventually learn enough about how nature works to be able to control it.	68	73	81	46	38	2.72	1.30	Anti-exemptionalism
If things continue on their present course, we will soon experience a major ecological catastrophe.	196	51	30	13	16	1.70	1.14	Eco-crisis

## Data Availability

The dataset to study would be available at request.

## Supplementary Materials

The following are available online at https://www.mdpi.com/article/10.3390/foods10123026/s1, Supplementary Materials: Survey questionnaires and Supplementary Table S1.
