# Supplementary material for: Perspectives and Attitudes towards the Functional and Safety Aspects of Seaweeds for Edible Applications in India
Source: Foods. 2021 Dec 6;10(12):3026. doi: 10.3390/foods10123026 (PMC8702041; doi:10.3390/foods10123026)
Supplement: Supplementary file 1 [file foods-10-03026-s001.zip › foods-1393084-supplementary.pdf]

Personal details:

1. Please select your age from the below mentioned brackets

   ◯ 15-20

   ◯ 21-30

   ◯ 31-50

   ◯ 50 and above

2. Please select the gender

   ◯ Male

   ◯ Female

   ◯ Transgender

3. Please select your present status

   ◯ Taking secondary or higher secondary education

   ◯ Student of graduation or post-graduation

   ◯ Self-employed

   ◯ Business

   ◯ Private sector service

   ◯ Government service

   ◯ Homecare/home maker

   ◯ Pursuing higher studies i.e. PhD/MS/MD

   ◯ Academic/Educational duties i.e. Teachers/Lab. assistant/Assistant Prof./Associate Prof./Professor

   ◯ Retired person

   ◯ Other

4. Please select your diet preference

   ◯ Strictly vegetarian

   ◯ Eggetarian and Vegetarian

   ◯ Strictly non-vegetarian

   ◉ Partly non-vegetarian

Fundamental understanding:

5. Any previous knowledge about seaweed based foods?

   ○ Yes ○ No

6. If yes, do you have knowledge about its nutritional contents?

   ○ Yes ○ No

7. If no, would you like to know more about this vegetarian alternative as a source of nutrition that has essential vitamin and mineral contents?

   ○ Yes ○ No

8. Seaweeds are a kind of marine plants (grow in seawater) and a rich source of antioxidants, essential minerals and dietary fibers. Do you agree they should be part of routine diet?

   ○ Strongly agree

   ○ Agree

   ○ May be

   ○ Disagree

   ○ Strongly disagree

9. If yes, what would be your preference in your diet?

   ☐As a part of maincourse

   ☐As a supplement/additive

   ☐As a soup

   ☐As a salad dressing

   ☐As a pickle

10. If no, pick up a reason

    ☐Issues with taste

    ☐Issues with smell

    ☐Due to the reliability of source

11. Are you aware that seaweeds are known to play role in extending life span of human beings?

    ○ Yes ○ No

12. Are you aware that seaweeds are part of routine diet in various Asian countries like Japan, Philippines, Indonesia and China?

   ○ Yes ○ No

13. Are you aware of restaurants that provide seaweed based snacks and meal in India?

   ○ Yes ○ No

14. Due to the contents of antioxidants, seaweeds are also used in cosmetic industries. Did you know such different uses of seaweeds?

   ○ Yes ○ No

15. Do you believe, for India having a long coastal area, seaweed cultivation can be an excellent opportunity for business?

   ○ Strongly agree

   ○ Agree

   ○ May be

   ○ Disagree

   ○ Strongly disagree

Safety concerns

16. Would you consider its source as an important factor prior to consumption?

   ○ Yes ○ No ○ May be

17. The cultivation of seaweed takes place along with the marine life forms, does that affects your preference as food?

   ○ Yes ○ No ○ May be

18. If yes, would you prefer to have an indoor-cultivated (grown using large seawater tanks) seaweed for food purpose.

   ○ Yes ○ No ○ May be

19. Do you believe there are risks associated with fresh/untreated use of seaweeds as food?

   ○ Yes ○ No ○ May be

20. Do you think that seaweed can be treated before consuming so that it does not pose any risk?

   ○ Yes ○ No ○ May be

21. Do you believe, the capability of seaweed to absorb heavy metals can negatively affect your health?

- Strongly agree
- Agree
- Require further analysis
- Disagree
- Strongly disagree

22. Do you believe, it may be associated with bacteria/fungi other microbes or pathogens that can cause diseases?

- Strongly agree
- Agree
- Require further analysis
- Disagree
- Strongly disagree

23. Foods with bioactive compounds are known to improve passive immunity, how much you agree to include such foods to be included in your routine diet?

- Strongly agree
- Agree
- Require further analysis
- Disagree
- Strongly disagree

24. Seaweeds are rich in bioactive compounds which have antiviral activities, do you believe it should be included in your routine diet?

- Strongly agree
- Agree
- Require further analysis
- Disagree
- Strongly disagree

25. Does the origin of bioactive compounds with antiviral/antibacterial activity matters for you prior its intake?

- Strongly agree
- Agree
- No preference
- Disagree
- Strongly disagree

Supplementary Table S1

SN	NEP-Perspective	Strongly Agree	Agree	Neutral	Disagree	Strongly Disagree
1	Humans have the right to modify the natural environment to suit their needs					
2	We are approaching the limit of the number of people the earth can support.					
3	When humans interfere with nature it often produces disastrous consequences					
4	Human ingenuity will ensure that we do not make the earth unlivable.					
5	Humans are severely abusing the environment					
6	The Earth has plenty of natural resources if we just learn how to develop them					
7	Plants and animals have as much right as humans to exist.					
8	The balance of nature is strong enough to cope with the impacts of modern industrial nations					
9	Despite our special abilities, humans are still subject to the laws of nature.					
10	The so-called “ecological crisis” facing humankind has been greatly exaggerated.					
11	The Earth is like a spaceship with very limited room and resources					
12	Humans were meant to rule over the rest of nature.					
13	The balance of nature is very delicate and easily upset.					
14	Humans will eventually learn enough about how nature works to be able to control it.					
15	If things continue on their present course, we will soon experience a major ecological catastrophe.					
